# The Camera Itself as a Calibration Pattern: A Novel Self-Calibration Method for Non-Central Catadioptric Cameras

**DOI:** 10.3390/s120607299

**Published:** 2012-05-30

**Authors:** Zhiyu Xiang, Bo Sun, Xing Dai

**Affiliations:** 1 Zhejiang Provincial Key Laboratory of Information Network Technology, Zhejiang University, No. 38, Zheda Road, Hangzhou 310027, China; 2 Department of Information Science and Electronic Engineering, Zhejiang University, No. 38, Zheda Road, Hangzhou 310027, China; E-Mails: sunbo51@gmail.com (B.S.); dexctor@gmail.com (X.D.)

**Keywords:** non-central catadioptric camera, self-calibration, mirror posture, mirror boundary, lens boundary

## Abstract

A novel and practical self-calibration method for misaligned non-central catadioptric cameras is proposed in this paper. Without the aid of any special calibration patterns in the scene, the developed method is able to automatically estimate the pose parameters of the mirror with respect to the perspective camera. First it uses the ellipse corresponding to the mirror boundary in the image to compute the possible solutions for mirror postures. With two pose candidates, thereafter we propose a novel selection method to find the correct solution by using the image of the lens boundary. The whole calibration process is automatic and convenient to carry out since nothing more than a single image acquired from the catadioptric camera is needed. Experimental results both on synthesized and real images prove our success.

## 1. Introduction

A catadioptric camera system usually consists of a revolutionary symmetric reflective mirror and a conventional perspective camera observing a scene reflected by the mirror. Such vision systems featuring the advantage of large field of view are being increasingly used in many applications [1–3], such as mobile robot navigation, video surveillance, virtual reality, outer space exploration and 3D reconstruction. Depending on whether they pose a single viewpoint [4,5], catadioptric cameras can be classified as central or non-central imaging systems.

Since camera calibration is a preliminary step in most applications, a variety of calibration methods for catadioptric systems have been reported. Currently most of these calibrations focus on central systems due to their popularity and relatively mature computing theories. However there are only a few combinations of mirror type and camera can have the opportunity to meet the single viewpoint requirement, which are insufficient for lots of applications. Furthermore when misalignment happens, all of the central catadioptric cameras become non-central, aside from when the mirrors are not the type in the central list [6]. Therefore non-central catadioptric systems are more general and the researches of calibration on them are of greater importance.

Calibration works on non-central systems can be divided into two categories. The first one focuses on non-central mirrors with unknown parameters and tries to model and calibrate them [5,7–9].The model is called caustic surface which represents the actual locus of the viewpoints. They use known light patterns [5] or known camera motion and point correspondence [7] to calculate the caustic. In [9], it was reported that three polarization images taken with different orientations could estimate the caustics of any combination of specular mirror and lens. During the calibration they assume the perspective camera is co-axial with the symmetric axis of the mirror. The second category [3,6,10,11] models the non-central system as a generalized camera where each pixel in the image corresponds to an incident ray reflected by a special point on the mirror. Micusik *et al.* [6] calibrated non-central cameras in two steps. First an approximate central camera is calibrated and then the non-central model was used to finish the 3D reconstruction. In [3] and [11] the forward projection model to calculate the reflective point on the mirror by non-iterative algorithms was proposed and used in motion estimation and 3D reconstruction. They all assume the coaxial mounting of the mirror and the perspective camera. Another major part of the researches in this category can deal with the misalignment between the camera and the mirror. Fabrizio *et al.* [10] presented a method which uses the internal and external mirror boundaries to calibrate the perspective camera and its posture relative to the mirror. In their configuration, a black needle was specially designed and mounted at the bottom of the mirror to provide the internal calibration circle. Mashita *et al.* [12] used the mirror boundary and a number of lines at infinity to estimate the mirror posture. Nonlinear optimization algorithms are commonly used in the calibration [11,13–15]. Strelow *et al.* [14] and Goncalves and Araujo [15] proposed methods for calibrating part and full parameters of the entire non-central camera systems by using bundle adjustment. They use preset calibration patterns in the scene and calculate the unknown parameters by minimizing the re-projection error. The accuracy of these methods mainly relies on the goodness of initial values because of nonlinear optimization. The majority of those misalignment-calibration methods can only deal with slight misalignment due to the assumptions they make in their algorithms. Caglioti [16] proposed a calibration algorithm for large off-axis non-central cameras by using line patterns. However they need the profile of the mirror to be seen in the image, which is not very practical in lots of cases. Recently Agrawal [3] extended their original work [17] to an off-axis forward projection model.

In this paper, we propose a novel self-calibration method for non-central catadioptric systems by using the camera itself as calibration pattern. Our method belongs to the second category listed above and is not subject to the constraint of slight misalignment. Like [10] and [12], we use the mirror boundary as the main reference pattern. However, unlike [10] and [12], where specially-designed needles or lines are used as extra calibration patterns, we do not need any extra calibration patterns apart from camera itself. We use the lens boundary of the perspective camera as extra condition. To our knowledge it is the first time that the calibration of anon-central system without using any extra calibration patterns apart from the camera itself is reported.

We focus on calibrating the relative pose between the perspective camera and the mirror. First, we use the ellipse-shaped mirror boundary image to estimate the four possible mirror postures based on Chen's extrinsic calibration method [18]. In order to remove the ambiguity of mirror posture, we then present a novel selection method making use of the imaged lens boundary to find the correct solution. Experiments conducted both on simulated data and real images confirm the performance of the proposed method.

In the following section the general model of non-central camera is briefly explained. After giving the algorithm idea in Section 3, Sections 4, 5, and 6 describe the three main steps of our algorithm in detail. Experimental results both on simulated data and real image are represented in Section 7. Finally, conclusions are given in the last section.

## 2. Camera Model

### 2.1. General Configuration of the Camera System

**Figure 1.**
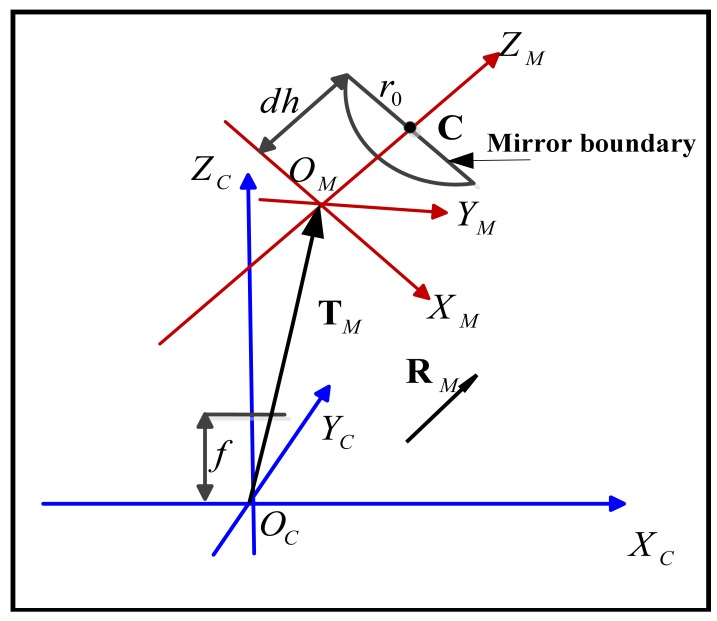
General configuration of the non-central catadioptric camera system.

Figure 1 shows the general configuration of a catadioptric camera system, where the camera and the mirror coordinate systems are denoted with the subscripts “*C*” and “*M*” respectively. Due to the misalignment the rigid body transformation between the two coordinate systems, *i.e.*, the rotation ***R****_M_* and translation **T***_M_*, drifts from the ideal configuration and makes the system non-central.

The full model of the non-central system should include the parameters of the mirror and the conventional perspective camera as well as the relative posture between the mirror and the camera. Generally the manufacture of the mirror can be fairly accurate and the deviation from the theoretic design could be very small. Meanwhile the intrinsic parameters of the perspective camera can also be computed in advance by some mature algorithms like the calibration toolbox from Jean-Yves Bouguet [19], and they do not change when misalignment of catadioptric system happens. Therefore we believe it is reasonable and valuable to find a good self-calibration method by computing the relative posture between the mirror and camera given their intrinsic parameters.

### 2.2. Perspective Camera Model

Let *X_C_* = (*X_C_,Y_C_,Z_C_*)*^T^* be the coordinates of a 3D point in the camera coordinate system and **ũ**=(*u,v*,1)*^T^* be the homogenous coordinates of the image point respectively, according to the pinhole model we have:

(1) $$s\tilde{\text{u}}={\text{KX}}_{c}$$

where *s* is a scale factor and **K** is the camera intrinsic matrix. For off-the-shelf camera the radial distortion in the image has to be removed before calibration with Equation (2):

(2) $$\{\begin{array}{c} x_{C}=x_{Cd}/\left(1+k_{1}\left(x_{C}^{2}+y_{C}^{2}\right)+k_{2}{\left(x_{C}^{2}+y_{C}^{2}\right)}^{2}\right)\\ y_{C}=y_{Cd}/\left(1+k_{1}\left(x_{C}^{2}+y_{C}^{2}\right)+k_{2}{\left(x_{C}^{2}+y_{C}^{2}\right)}^{2}\right) \end{array}$$

where *k*_1_, *k*_2_ are radial distortion coefficients (*x_c_, y_c_*) and (*x_Cd_, y_Cd_*) are the undistorted and distorted normalized image coordinates, respectively. The intrinsic parameters of the perspective camera can be calibrated independently and are assumed known in advance throughout the paper.

## 3. Algorithm Idea

The idea of the calibration algorithm will now be described. Before calibration a calibrating image should be acquired from the catadioptric camera. Different from most of the existing calibration methods, there is no any special calibration pattern in the environment. The only requirement for the image is the mirror boundary and the lens boundary or at least part of them should be clearly visible in the image, which is true for most of the catadioptric cameras. Now it is required to determine the mirror posture relative to the camera coordinate system. The steps of the algorithm are as follows.

1. Image preprocessing and robust ellipse extracting. The images of the mirror and lens boundary can be extracted and fitted with ellipses, which actually encodes the mirror posture (Section 6).
2. Mirror posture computing based on the imaging ellipse of the mirror boundary. It is a non-iterative method and finally two posture candidates are obtained (Section 4).
3. Correct posture selecting based on the image of lens boundary. During the selection process, the unknown position of the real lens boundary relative to the optical center should also be estimated simultaneously (Section 5).

The steps of this algorithm will be described in detail in the subsequent sections of the paper. The main subject of this paper comprises the last two steps of this algorithm, which will be described first. The first step is of peripheral interest, and a description of the robust method used for automatic ellipse extracting and fitting will be postponed to a later section.

## 4. Computing Mirror Posture Candidates

As show in Figure 1, the mirror posture can actually be represented by the transformation between the camera coordinate system *O_C_* − *X_C_Y_C_Z_C_* and the mirror coordinate system *O_M_* − *X_M_Y_M_Z_M_*. With intrinsic parameters of the perspective camera in hand, the next step is to compute the possible solutions of mirror postures by using the image of the mirror boundary. We apply Chen's method [18] to accomplish this. Considering a camera coordinate system *O_C_* − *X_C_Y_C_Z_C_* that the origin *O_C_* is the optical center and the *Z_C_*-axis is the optical axis asshown in Figure 1, the imaged mirror boundary can be described as an ellipse with the following form:

(3) $$Au^{2}+Bv^{2}+2Cu+2Dv+2\text{Euv}+F=0$$

The quadratic form of Equation (3) is:

(4) $${\tilde{\text{u}}}^{\text{T}}{\text{Q}}_{I}\tilde{\text{u}}=0$$

where **ũ**=(*u,v*,1)*^T^* represents the image coordinates of the points in the ellipse and:

(5) $${\text{Q}}_{I}=\left[\begin{array}{ccc} A & E & C\\ E & B & D\\ C & D & F \end{array}\right]$$

Substituting **ũ** in Equation (4) by Equation (1), an oblique ellipse cone in the camera coordinate system is obtained as follows:

(6) $$s^{2}{\text{X}}_{C}^{T}{\text{Q}}_{C}{\text{X}}_{C}=0$$

where:

(7) $${\text{Q}}_{C}={\text{K}}^{\text{T}}{\text{Q}}_{I}\text{K}.$$

As shown in Figure 1, a mirror boundary coordinate system *O_mb_* − *X_mb_Y_mb_Z_mb_* (the subscript “*mb*” stands for “mirror boundary”) is defined where the origin *O_mb_* overlapswith the optic center *O_C_* and *Z_mb_*-axis parallel with the unitnormal vector of the supporting plane of the circle to beviewed. The circle of mirror boundary centered at **C***_mb_*=(*x*_0_, *y*_0_, *z*_0_)*^T^* on *Z_mb_*=*Z*_0_ plane with known radius *r*_0_ can be described as:

(8) $${\text{X}}_{mb}^{T}{\text{MX}}_{mb}=0$$

where:

(9) $$\text{M}=\left[\begin{array}{ccc} 1 & 0 & -x_{0}/z_{0}\\ 0 & 1 & -y_{0}/z_{0}\\ -x_{0}/z_{0} & -y_{0}/z_{0} & \left(x_{0}^{2}+y_{0}^{2}-r^{2}\right)/z_{0}^{2} \end{array}\right]$$

and **X***_mb_* is the mirror boundary points represented in the mirror boundary coordinate system.

Based on the definition of the two coordinate systems, only a rotation **R***_M_* exists between the camera and mirror boundary coordinate system and the relationship can be expressed as:

(10) $${\text{X}}_{C}={\text{R}}_{M}{\text{X}}_{mb}$$

Integrating Equations (6), (8) and (10) we have:

(11) $$s^{2}{\text{R}}_{M}^{T}{\text{Q}}_{C}{\text{R}}_{M}=\text{M}$$

To solve the above equation, first we convert **Q**_c_ into a diagonal matrix by eigenvalue decomposition:

(12) $${\text{Q}}_{C}=\text{V}\Lambda {\text{V}}^{T}$$

where Λ = *diag*{*λ*_1_,*λ*_2_,*λ*_3_}, **V** = (*v*_1_,*v*_2_,*v*_3_). From Equations (11) and (12), we get:

(13) $$s^{2}{\text{R}}^{T}\Lambda \text{R}=\text{M}$$

where **R** = **V***^T^***R***_M_*. We assume *λ*_1_*λ*_2_ > 0 and |*λ*_1_| > |*λ*|_2_ Solving **R***^T^***R** = **I** and Equation (13), we finally get **R** according to [18], where θ is a free variable, *S*_1_ and *S*_2_ are undetermined signs, and $g_{1}=\sqrt{\left(\lambda_{2}-\lambda_{3}\right)/\left(\lambda_{1}-\lambda_{3}\right)},g_{2}=\sqrt{\left(\lambda_{1}-\lambda_{2}\right)/\left(\lambda_{1}-\lambda_{3}\right)}:$

(14) $$\text{R}=\left[\begin{array}{ccc} g_{1}\cos\theta & S_{1}g_{1}\sin\theta & S_{2}g_{2}\\ \sin\theta & -S_{1}\cos\theta & 0\\ S_{1}S_{2}g_{2}\cos\theta & S_{2}g_{2}\sin\theta & -S_{1}g_{1} \end{array}\right]$$

And from Equations (12), (13) and (14), the rotation from the mirror boundary coordinate to the camera coordinate is:

(15) $${\text{R}}_{M}=\text{VR}$$

**Figure 2.**
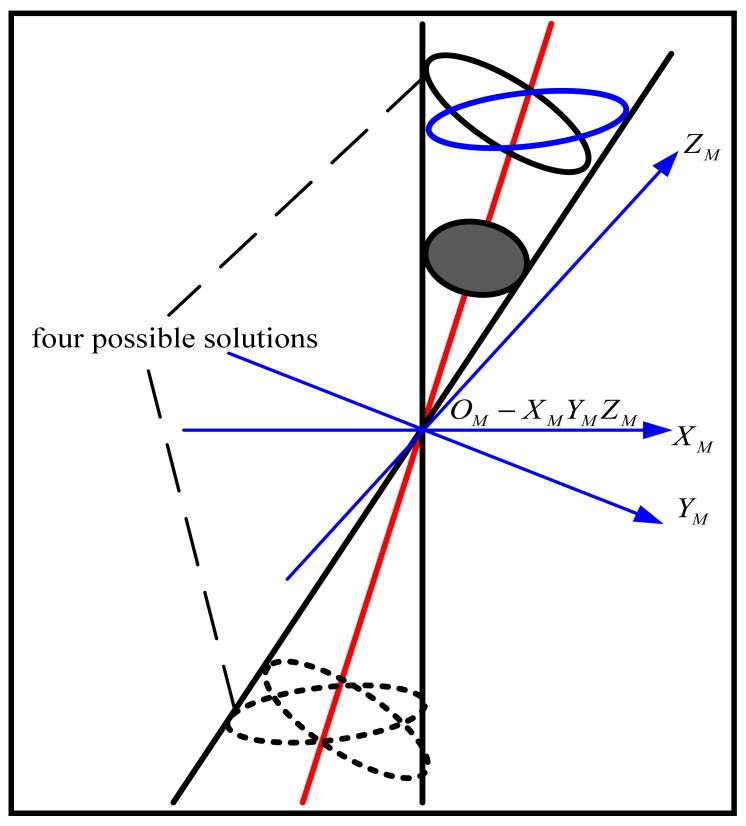
Illustration of four possible solutions.

Finally we can describe the center of the circle and unit normal vector of the supporting plane in the camera coordinate system by the following expression:

(16) $$\{\begin{array}{c} z_{0}=S_{3}\lambda_{2}\frac{r_{0}}{\sqrt{-\lambda_{1}\lambda_{3}}}\\ {\text{n}}_{C}={\text{R}}_{M}{\left(0,0,1\right)}^{T}=\text{V}{\left(S_{2}g_{2},0,-S_{1}g_{1}\right)}^{T}\\ {\text{C}}_{C}={\text{R}}_{M}{\text{C}}_{mb}=z_{0}\text{V}{\left(S_{2}g_{2},0,-S_{1}g_{1}\frac{\lambda_{1}}{\lambda_{2}}\right)}^{T} \end{array}$$

where *S*_3_ is an undetermined sign. Equation (16) gives a total of four sets of possible solutions although some of them are not physically possible, as shown in Figure 2.

In the mirror coordinate system *O_M_* − *X_M_Y_M_Z_M_*, being the central symmetric axis of the mirror, the *Z_M_*-axis is parallel to the vector **n***_C_* while the origin *O_M_* does not overlap with the optical center. The translation between the mirror and the camera coordinate system is obtained by Equation (17), where *dh* is the distance from the mirror boundary center to *O_M_*.

(17) $${\text{T}}_{M}={\text{C}}_{c}-{\text{R}}_{M}{\;(0,0,dh)}^{T}$$

Constraining **n***_C_* points back towards the camera and ***C****_C_* lies in front of the camera as follows:

(18) $$\{\begin{array}{c} z_{0}>0\\ {\text{n}}_{C}•{\left(0,0,1\right)}^{T}>0\\ {\text{C}}_{C}•{\left(0,0,1\right)}^{T}>0 \end{array}$$

we can get two candidate solutions $\left\{{\text{R}}_{M}^{i},{\text{T}}_{M}^{i}\right\}\left(i=1,2\right)$ of the mirror posture which are both physically possible.

## 5. Mirror Pose Selection

Obviously more constraints are necessary to finally select the correct solution from the obtained posture candidates. In order for the calibration method to be independent of any special designed calibration patterns, we propose a novel method to achieve this by using the image of the lens boundary.

In the calibrating image, the lens boundary of the camera can also be easily observed in the center part of the image. The observed lens boundary in the image, whose size and position depends on the posture and the shape of the mirror, can be represented by a closed curve. Normally the curve is difficult to describe strictly by some special type of conics. For the revolutionary symmetric mirrors used in the catadioptric system, we find fitting the curve into an ellipse is accurate enough for the pose selection purpose, as we will see in the experiments. Theoretically, given the 3D position and the size of the lens boundary in the camera coordinate system, the correct posture can be selected by checking the similarity between the “observed” and the “predicted” lens boundary. There are two ways to define the “observed” and the “predicted” boundaries, each of which corresponds to checking the similarity under 2D image plane or 3D camera coordinate. In the 2D image plane, the “observed” one is the real ellipse we obtained and the “predicted” one is an ellipse computed by projecting the lens reflection on the mirror to the image plane. That is what our previous work has done [20] and it needs to calculate the reflective point on the mirror for each point on the lens boundary. The calculation has to be finished by a nonlinear optimization process and is very time-consuming. Here we propose a better way to check the similarity, where the “observed” is the real lens boundary in 3D camera coordinate and the “predicted” is the intersecting curves of a “cutting plane” with the reflective “cone” composed of the incident rays from the lens boundary. The “cutting plane” is actually the plane where the lens boundary lies in the camera coordinate system. For each pixel in the imaged lens boundary, the corresponding mirror points can be computed straightly and consequently the reflective “cone” can be obtained. Therefore this algorithm is much more effective.

However, in practice not all of the parameters of the lens boundary are known. Among those unknown the most important are the position parameters. Only the radius of the lens boundary can be found in the data sheet of the lens. By reasonably assuming the plane where the lens boundary lies be parallel with the X-Y plane of the camera coordinate system, the distance of the plane to the optical center is still left as an unknown. Therefore we need to estimate the distance of the lens boundary and select the correct mirror posture solution simultaneously.

Apparently this selection method can be applied to any type of the mirror as long as the mirror parameters are known. For simplicity we take the common hyperboloidal mirror as an example to explain the computing process.

The hyperboloidal mirror can be expressed by Equation (19):

(19) $$\frac{Z^{2}}{a_{m}^{2}}-\frac{X^{2}+Y^{2}}{b_{m}^{2}}=1$$

Transforming Equation (19) into the canonical form yields:

(20) $${\text{X}}_{\text{M}}^{\text{T}}{\text{Q}}_{\text{M}}{\text{X}}_{\text{M}}=0$$

where:

(21) $${\text{Q}}_{M}=\left[\begin{array}{cccc} 1 & 0 & 0 & 0\\ 0 & 1 & 0 & 0\\ 0 & 0 & -b_{m}^{2}/a_{m}^{2} & 0\\ 0 & 0 & 0 & b_{m}^{2} \end{array}\right]$$

and **X***_M_*=[*X,Y,Z*,1]*^T^* the homogeneous coordinates of the mirror points. Then the quadric mirror can be expressed in the camera coordinate system as:

(22) $${\text{Q}}_{C}^{i}={\left({\text{H}}^{i}\right)}^{-T}{\text{Q}}_{M}{\left({\text{H}}^{i}\right)}^{-1}={\left[\begin{array}{cc} {\text{R}}_{M}^{i} & {\text{T}}_{M}^{i}\\ 0 & 1 \end{array}\right]}^{-T}\cdot \left[\begin{array}{cccc} 1 & 0 & 0 & 0\\ 0 & 1 & 0 & 0\\ 0 & 0 & -b_{m}^{2}/a_{m}^{2} & 0\\ 0 & 0 & 0 & b_{m}^{2} \end{array}\right]\cdot {\left[\begin{array}{cc} {\text{R}}_{M}^{i} & {\text{T}}_{M}^{i}\\ 0 & 1 \end{array}\right]}^{-1}$$

**Figure 3.**
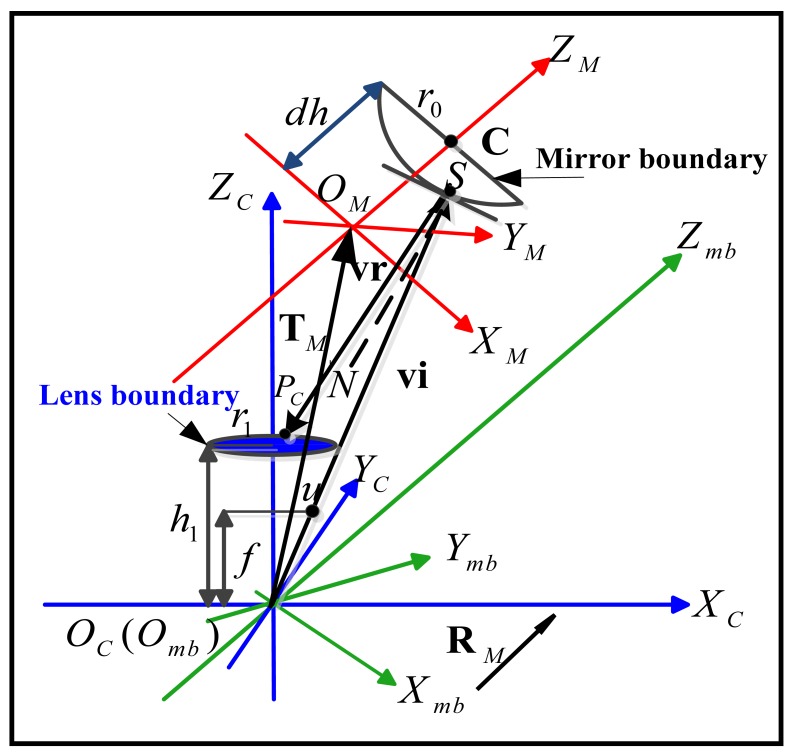
Relative position among lens boundary and three other coordinate systems: camera (blue), mirror (red) and mirror boundary (green).

Given the mirror parameter ${\text{Q}}_{C}^{i}(i=1,2)$, the next step is to obtain a reflective cone emitting from the mirror corresponding to the image of lens boundary. Due to the nonlinearity of the mirror shape, it's difficult to obtain a linear matrix form for the reflective cone. However as shown in Figure 3, the cone can be represented by a set of discrete reflective rays **vr** emitting from the mirror pointS, where each ray **vr***_ij_* corresponds to a sampling point **u***_j_*(*j* = 0,1,2,…,*n*) in the imaged lens boundary.

In camera coordinates, from the perspective projection model, we have:

(23) $${\tilde{\text{u}}}_{ij}={\tilde{\text{u}}}_{j}=\frac{1}{\lambda }\text{K}\left[\text{I}|0\right]{\text{S}}_{C}^{ij}$$

Therefore the incident ray ${\text{v}}_{ij}={\left[v_{1}^{ij},v_{2}^{ij},v_{3}^{ij}\right]}^{T}$ corresponds to *u_ij_* can be parameterized as:

(24) $${\text{v}}_{ij}=\lambda {\text{K}}^{-1}{\tilde{\text{u}}}_{ij}=\lambda {\text{K}}^{-1}{\tilde{\text{u}}}_{j}$$

With the normalized incident ray ${\bar{\text{v}}}_{ij}=\frac{{\text{v}}_{ij}}{\left\|{\text{v}}_{ij}\right\|}$ in hand, the reflective mirror point $S_{C}^{ij}(\lambda )={\left[\lambda {\bar{\text{v}}}_{ij}^{T}\;1\right]}^{T}$ can be determined by the factor *λ* which satisfies Equation (25):

(25) $${\left({\text{S}}_{C}^{ij}\left(\lambda \right)\right)}^{T}{\text{Q}}_{C}^{i}{\text{S}}_{C}^{ij}\left(\lambda \right)=0$$

Expanding Equation (25) yields a quadric equation about *λ*. By imposing a positive depth constraint the correct root *λ*_0_ can be selected from the solution and hence ${\text{S}}_{C}^{ij}$ can be determined. The normal vector to the quadric mirror at the reflective point is given by the first three coordinates of the tangent plane $\Pi_{N}^{ij}={\text{Q}}_{C}^{ij}{\text{S}}_{C}^{ij}$. The normalized normal vector is thus given by:

(26) $${\text{n}}_{ij}=\frac{[{\text{I}}_{3}|0]{\text{Q}}_{C}^{i}{\text{S}}_{C}^{ij}}{\left\|[{\text{I}}_{3}|0]{\text{Q}}_{C}^{i}{\text{S}}_{C}^{ij}\right\|}$$

where **I**_3_ is a 3×3 identity matrix. The reflective vector **vr***_ij_* can then be obtained according to the law of reflection:

(27) $$\text{v}{\text{r}}_{ij}={\bar{\text{v}}}_{ij}-2({\bar{\text{v}}}_{ij}•{\text{n}}_{ij}){\text{n}}_{ij}$$

Now we have two set of rays **vr***_ij_*(*i*=1,2; *j*=1,2,…,*n*) which represent two reflective cones corresponding to the two posture candidates respectively. As shown in Figure 3, the lens boundary with radius *r*_1_ lies ahead of the optical center *O_C_* with distance *h*_1_. Two sets of intersecting points ${\text{P}}_{C}^{ij}$ can be obtained by intersecting the rays **vr***_ij_* with the “cutting plane” at a distance *h*_1_ to *O_C_*. Writing ${\text{S}}_{C}^{ij}={\left[S_{1}^{ij},S_{2}^{ij},S_{3}^{ij},1\right]}^{T}$ and $\text{v}{\text{r}}_{ij}={\left[vr_{1}^{ij},vr_{2}^{ij},vr_{3}^{ij}\right]}^{T}$, ${\text{P}}_{C}^{ij}$ can be computed by Equation (28):

(28) $${\text{P}}_{C}^{ij}=\lambda_{0}{\bar{\text{v}}}_{ij}^{T}+\frac{h_{1}-S_{3}^{ij}}{vr_{3}^{ij}}\text{v}{\text{r}}_{ij}$$

Taking *h*_1_ as an unknown variable, one dimensional searching on *h*_1_'s supporting region $\left[0,\sqrt{a_{m}^{2}+b_{m}^{2}}\right]$ could be carried out. For each possible combination of posture labeled *i* and *h*_1_, one set of ${\text{P}}_{C}^{ij}$ can be obtained by Equation (28). The set of the points can be fitted into an ellipse whose center $O(i,h_{1})={\left[O_{x}^{\left(i,h_{1}\right)},O_{y}^{\left(i,h_{1}\right)},h_{1}\right]}^{T}$ and the length of the major axis *a*(*i*, *h*_1_) and minor axis *b*(*i*, *h*_1_) are all the functions of variables *i* and *h*_1_ and can all be computed, respectively. Obviously the correct *i* and *h*_1_ should result in a circle with radius *r*_1_ and central coordinates (0,0,*h*_1_)*^T^*. Therefore it is reasonable to construct an error function *E* measuring the difference between the intersecting ellipse and the real lens boundary as follows:

(29) $$E\left(i,h_{1}\right)=\sqrt{{\left(O_{x}^{\left(i,h_{1}\right)}\right)}^{2}+{\left(O_{y}^{\left(i,h_{1}\right)}\right)}^{2}}+\sqrt{{\left(a\left(i,h_{1}\right)-r_{1}\right)}^{2}+{\left(b\left(i,h_{1}\right)-r_{1}\right)}^{2}}\left(i=1,2;h_{1}\in \left[0,\sqrt{a_{m}^{2}+b_{m}^{2}}\right]\right)$$

In Equation (29) the first term and the second term of *E* represent the central position error and the radius error respectively. Finally, the correct *i* and *h*_1_ can be obtained byminimizing the function, that is:

(30) $$\left(i^{*},h_{1}^{*}\right)=\underset{i,h_{1}}{\arg\min E(i,h_{1})}$$

The function *E* is difficult to differentiate analytically, therefore a derivative-free optimizer is preferred. The downhill simplex method is a good candidate for this type of optimization [21].

## 6. Robust Ellipse Extracting and Fitting

In order to estimate the mirror posture, ellipses of the mirror and lens boundaries in the image need to be extracted accurately. Given a calibrating image, we propose a robust method to finish the ellipse extraction task automatically. First the Canny operator is applied to obtain an edge image. Then by using two Regions of Interest (ROIs) where the mirror boundary and lens boundary should appear respectively, most of the edge pixels outside the ROIs are removed. Meanwhile edge pieces with small length are also deleted from the image. After that, we do iterative least mean square ellipse fitting to all the remaining pixels within each ROI until convergence. As can be seen from Figure 10, the ideal mirror and lens boundary in the image appear to be the outmost and inmost elliptical contour. For mirror boundary, the initial fitted ellipse always sits inside the real boundary due to the existence of non-boundary pixels. Therefore during each iteration those edge pixels staying inside the fitted ellipse are removed so as to “expand” the fitted ellipse in the next iteration. A similar process is applied to the lens boundary except that a “shrinkage” strategy is used. The iteration continues until the fitting error is less than a threshold. To further improve the fitting accuracy, 5-point RANSAC ellipse fitting is applied to the rest edge pixels and the final optimal ellipse parameters can be obtained [22,23]. The advantage of this technique is that it can automatically extract the ellipse and obtain the ellipse parameters with high accuracy.

## 7. Experiments

To verify the proposed self-calibration method, some experiments based on simulation data and real images were carried out.

### 7.1. Experiment with Simulation Data

Based on the simulated camera configuration listed in Table 1, the synthesized imaging ellipses of the mirror and lens boundaries can be easily generated for calibration, respectively.

The calibration results are summarized in Table 2. They show that the calibration method is effective and the proposed selection method does find the correct pose solution and the height h_1_.

**Figure 4.**
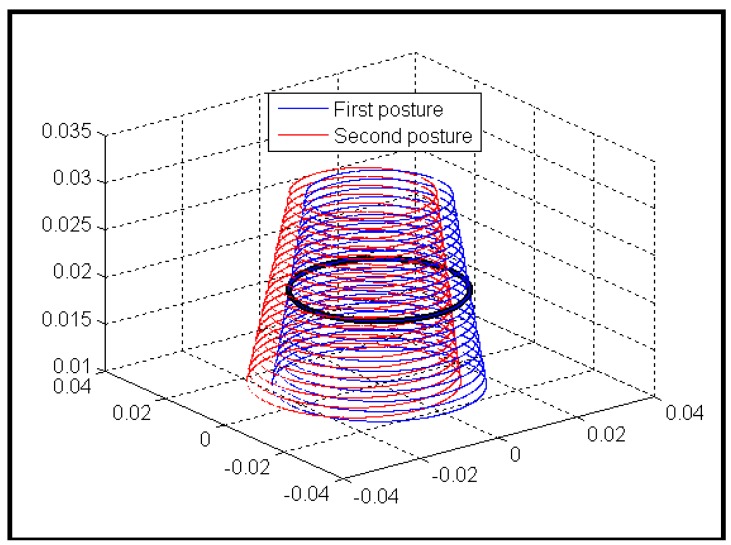
Illustration of the actual (black) and the two sets of the predicted (red and blue) lens boundaries.

**Figure 5.**
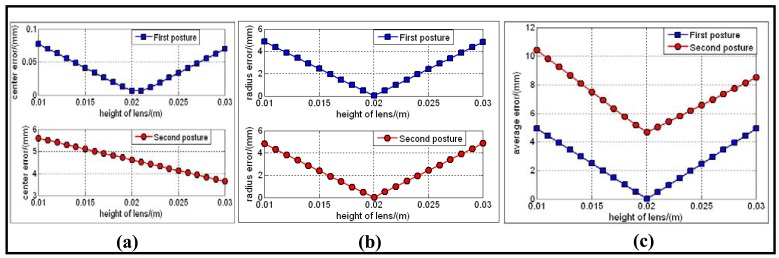
(**a**) Central position error with respect to*h*_1_. (**b**) Radius error with respect to *h*_1_. (**c**) Combined error E with respect to *h*_1_.

Figure 4 shows the typical variance of the position and the size of the two predicted lens boundaries with respect to the actual one within *h*_1_'s supporting region. Figure 5(a–d) shows the central error, radius error, and combined average error between the predicted and the actual lens boundary respectively. From the two figures we can confirm that the minimum average error is reached when *h*_1_ is near 0.02 m, and the correct pose solution has been selected out.

**Figure 6.**
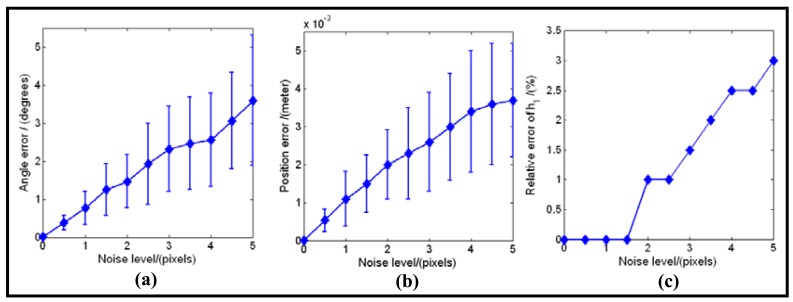
Calibration errors w.r.t. noise level: (**a**) direction error of the normal vector.(**b**) position error of the mirror center. (**c**) relative percentage error of the detected *h*_1_.

The results listed in Table 2, Figures 4 and 5 did not consider the noises which usually exist in the process of the boundary imaging, edge extraction and ellipse fitting. To see the robustness of the calibration method in the presence of noise, we added zero-mean Gaussian noise with standard deviation *σ* to the sampled points on the image of mirrorboundary and lensboundary. *σ* varies from 0 to 5 pixels in 0.5 pixel steps. For each noise level, the mirror posture and *h*_1_ areestimated by our algorithm. The difference between the estimated parameters and the ground truth were recorded as an error measurement. The resulting error of **n***_C_* (angles, unitin degree), the error of **C***_C_* (Euclidean distance, unit in meters) and the relative error of the detected *h*_1_ are shownin Figure 6. In all of the test cases, the calibration algorithm produced stable posture choices and the solution closer to the ground truth was correctly found. The results show that the proposed algorithm is robust in the condition of different noise levels.

### 7.2. Experiment with Real Images

#### 7.2.1. Catadioptric Camera Setup

**Figure 7.**
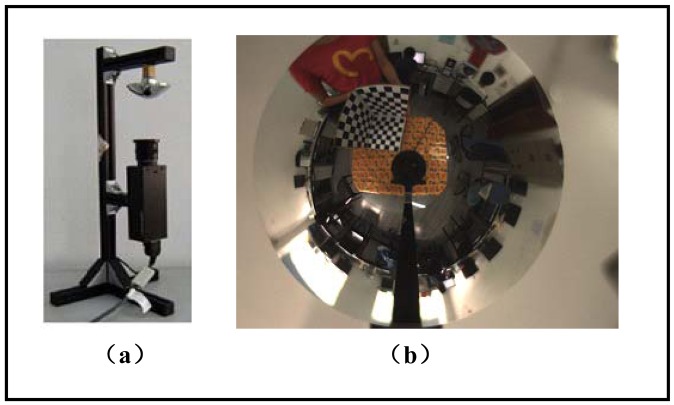
(**a**) The NEOVISION catadioptric camera system used in our experiment. (**b**) The omnidirectional image acquired by the system.

The catadioptric camera system we used for real data experiment is made by NEOVISION and is shown in Figure 7. It consists of a H3S hyperbolic mirror and a Sony XCD-SX910CR camera and was originally made as a central single viewpoint camera. More specifications of the system are listed in Table 3.We deliberately changed the relative position between the mirror and the camera, making it bias from the factory configuration. Therefore it was not a central camera again and actually became a new non-central catadioptric system. The intrinsic parameters of the conventional camera XCD-SX910CR are listed as follows: *f_x_* = 1455.07, *f_y_* = 1459.51, *k_s_* = 0, *u*_0_ = 639.2, *v*_0_ = 482.2, the image resolution is 1,280 × 960. The radius of the lens boundary is: *r*_1_=0.0185*m*.

#### 7.2.2. Ellipse Extracting and Fitting Results

**Figure 8.**
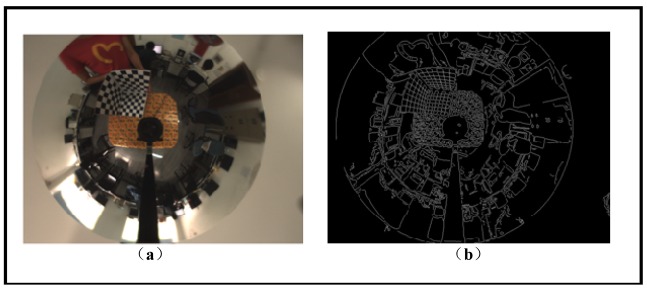
(**a**) Undistorted omnidirectional image. (**b**) Canny detection result.

**Figure 9.**
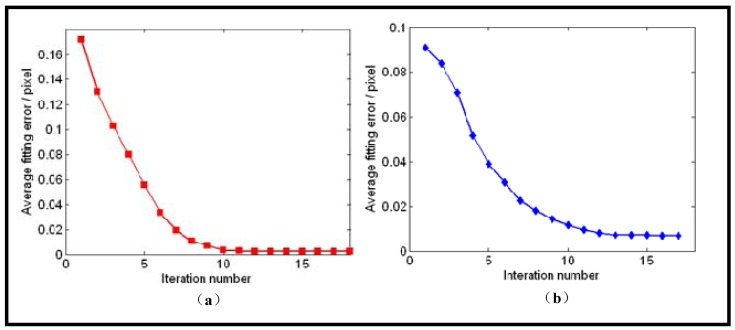
(**a**) Average fitting error of mirror boundary with respect to the fitting iterations. (**b**) Average fitting error of lens boundary with respect to the fitting iterations.

Figure 8 shows the calibration image and its canny detection result. Figure 9(a,b) shows the average fitting error of two boundaries with respect to the iteration number, respectively. It demonstrates that our iterative ellipse fitting process can refine the boundaries and quickly leads to convergence.

**Figure 10.**
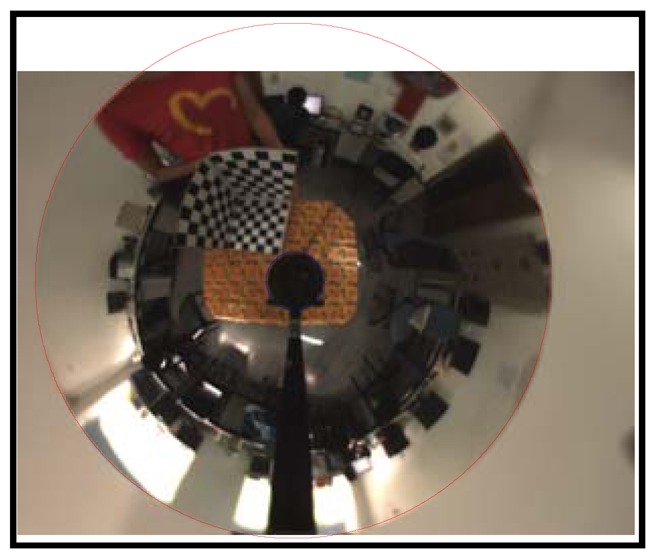
Final result of ellipse fitting.

After using the remaining edge pixels for 5-point RANSAC, we get the final extracted and fitted ellipses, as shown in Figure 10. The ultimate average fitting errors of mirror and lens boundaries are 0.0022 and 0.0071 pixels, respectively.

#### 7.2.3. Real Image Calibration

As we have the ellipse parameters of the mirror and the lens boundaries in hand, we apply our calibration algorithm to Figure 10. The final solution obtained is listed in Table 4. The resulting combined error between the actual and predicted lens boundary was 2.263 mm, which is very satisfying considering the existence of imaging noises.

### 7.3. Applications

#### 7.3.1. Image Transformation

In this section, we evaluate the performance of our calibration method by image transformation. Since our catadioptric camera system does not maintain the single viewpoint characteristic, we cannot transform the whole omnidirectional image into a perspective one, but it is still possible to transform a patch of acquired image by assuming an approximate single viewpoint.

**Figure 11.**
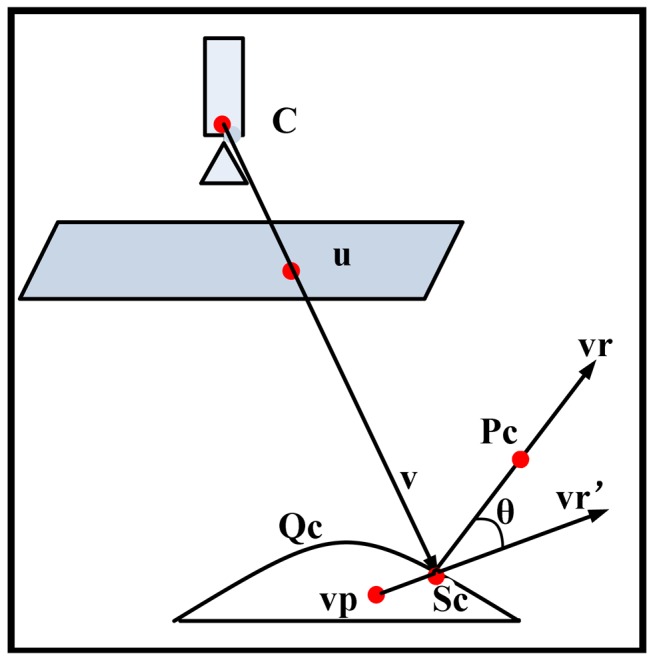
Illustration of finding an approximate single viewpoint.

As shown in Figure 11, we try to find a virtual single viewpoint *vp* by minimizing the sum of the angle error between each real ray *vr* and the virtual ray *vr'* originated from the viewpoint *vp*, as expressed in Equation (31):

(31) $$\text{vp}*=\underset{\text{vp}}{\arg\min}\left\{\sum_{i}\frac{180}{\pi }\text{arc}\cos\left(\frac{vr_{i}•v{r^{\prime }}_{i}}{\left\|vr\right\|\left\|v{r^{\prime }}_{i}\right\|}\right)\right\}.$$

**Figure 12.**
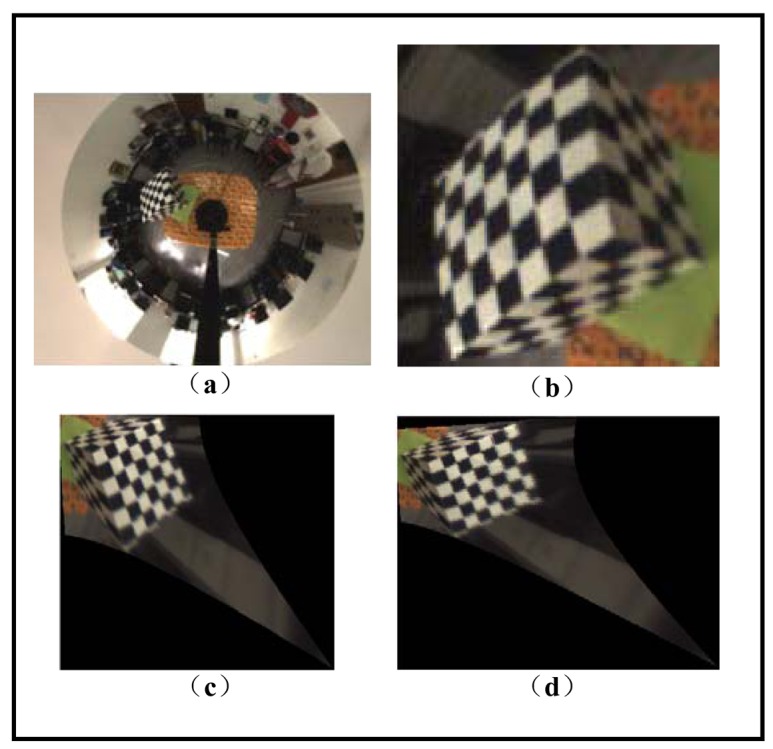
(**a**) Original omnidirectional image. (**b**) Sub-image area to be converted. (**c**) Perspective image produced by ideal configuration. (**d**) Perspective image produced by our calibration results.

Figure 12(a,b) shows the original omni-directional image and the zoom-in view of the sub-image area to be converted, respectively. Figure 12(c) shows the perspective image transformed by the ideal mirror posture from factory configuration. Figure 12(d) shows the perspective image transformed from our calibration results. In the images one can easily observe the difference caused by the change of viewpoint, which corresponds to the bias of the “real” viewpoint from the ideal one. Other rectifying effects such as removal of distortion of the lines are comparable and not very distinct due to the low resolution of the image.

#### 7.3.2. 3D Reconstruction

**Figure 13.**
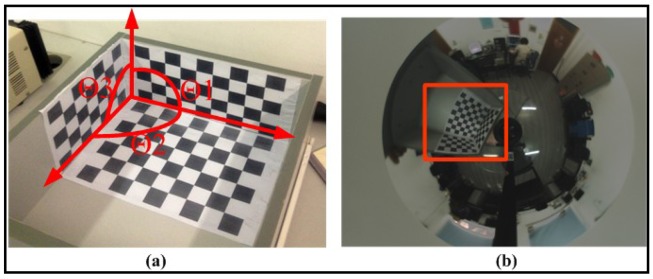
(**a**) Trihedron used in experiment. (**b**) Real image with trihedron in the scene.

To further verify the performance of our calibration algorithm quantitatively, we employed a trihedral object composed of three orthogonal checker patterns of known size (Figure 13(a)) and computed the angles between normal vectors of each checkerboard plane. First a sub-image containing the trihedral object was transformed into a perspective one using the method described above. Then a traditional camera calibration toolbox [19] was used to compute the normal directions of the three checkerboard planes respectively. Finally the three angles Θ1, Θ2 and Θ3, as shown in Figure 13(a) can be obtained.

Two different non-central configurations are designed for the experiment. The first configuration is denoted as “slightly non-central” which means the mirror focus is only biased a little from the ideal position. The second configuration is denoted as “medium non-central” which has several centimeters in the translation and several degrees in the rotation away from the ideal case. The posture parameters from the default factory configuration, Mei's calibration method [24] and our method are used for computing the angles, respectively. In each configuration 10 images with the trihedral object at different positions around the camera are acquired and the average values of the computed angles are recorded in Table 5. The three angles should all be 90 degrees in an ideal situation. From the table, we can see that angles calculated from our calibration algorithm are better than results from the default factory configuration in both configurations. For the slightly non-central case the results from our method and Mei's are comparable, while for the second case our method shows superior performance than Mei's. The reason is Mei's method is only designed for the central camera while our method can deal with the central and non-central situation equally.

## 8. Conclusions

A novel self-calibration method for non-central catadioptric cameras is proposed in this paper. We use the mirror boundary in the image to obtain the possible mirror posture candidates, and then select the correct solution by using the image of the lens boundary. In the implementation stage we also presented a robust ellipse extraction algorithm based on iterative outlier rejection followed by RANSAC. Both the computer simulation and real data have been used to test the proposed technique, and very satisfying results have been obtained. The calibration method is not subject to the constraint of slightly non-central misalignment and is able to calibrate the non-central camera in a single image using only the catadioptric camera itself. This also makes the method qualified for on-the-fly calibration processes and is particularly beneficial for the situation where no calibration patterns are available, such as off-road and planet robot navigation.

## Figures and Tables

**Table 1.** Simulated camera configuration.

**Intrinsic Parameters**	**Focal Length (pixel)**	*f_x_* = *f_y_* = 1500
	**Skew Coefficient**	*k_s_* = 0
	**Principal Points**	*u*_0_ = 640, *v*_0_ = 480

**Mirror Parameters**	**Mirror Boundary radius (m)**	*r*_0_ = 0.028
	**Major axis (m)**	*a_m_* = 0.028
	**Minor axis (m)**	*b_m_* = 0.023

**Lens Parameters**	**Lens Boundary radius (m)**	*r_1_* = 0.018
	**Height From Optical (m)**	*h_1_* = 0.02

**Mirror Posture**	**Mirror Center to** *O _M_* **(m)**	*dh* = 0.0425
	**Normal vector**	**n***_C_*=(0.0349, −0.0523, 0.9980)*^T^*
	**Mirror Center (m)**	**C***_C_*=(0.0002, 0.0005, 0.083)*^T^*

**Table 2.** Calibration results with simulated data.

**Parameters**	**Solution**

**Mirror Center *C****_C_* **(m)**	**C***_C_*_1_=(0.0002, 0.0005, 0.0830)*^T^*, **C***_C_*_2_=(0.0008, −0.0005, 0.0830)*^T^*

**Translation T***_M_* **(m)**	T*_M_*_1_=(−0.0013, 0.0027, 0.0406)*^T^*, **T***_M_*_2_=(0.0017, −0.0027, 0.0406)*^T^*

**Rotation R***_M_*	${\text{R}}_{M1}=\left[\begin{array}{ccc} -0.6299 & -0.7759 & 0.0349\\ 0.7742 & -0.6308 & -0.0523\\ 0.0626 & -0.0059 & 0.9980 \end{array}\right],{\text{R}}_{M2}=\left[\begin{array}{ccc} -0.6305 & -0.7758 & -0.0235\\ 0.7742 & -0.6308 & -0.0524\\ -0.0555 & 0.0149 & 0.9983 \end{array}\right]$

**Height *h*_1_ (m)**	*h*_1_=0.02

**Calibration Result**	**T***_M_*_1_,**R***_M_*_1_,*h*_1_

**Table 3.** H3S hyperbolic mirror specification.

**Type**	hyperbolic	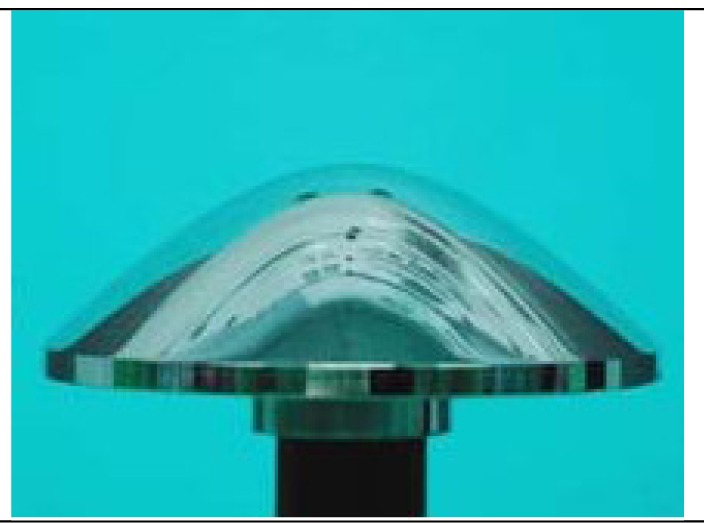
**Major Axis**	28.0950 mm	
**Minor Axis**	23.4125 mm	

**Table 4.** Calibration results of real image.

**Parameters**	**Solution**

**Mirror Center C***_C_* **(m)**	**C***_C_* = (0.0012, −0.0060, 0.0796)*^T^*

**Translation T***_M_* **(m)**	**T***_M_* = (0.0002, −0.0065, 0.0339)*^T^*

**Rotation R***_M_*	${\text{R}}_{M}=\left[\begin{array}{ccc} 0.0756 & -0.9969 & 0.0216\\ 0.9971 & 0.0758 & 0.0105\\ -0.0121 & 0.0207 & 0.9997 \end{array}\right]$

**Height *h*_1_ (m)**	*h*_1_ = 0.013

**Table 5.** Angles between normal of the planes in trihedron.

**Methods**	**Slightly non-central/degrees**			**Medium non-central/degrees**		

	**Θ1**	**Θ2**	**Θ3**	**Θ1**	**Θ2**	**Θ3**

**Factory Configuration**	85.37	96.58	83.65	93.41	107.99	91.33
**Mei's Method**	87.65	91.12	88.70	92.01	62.54	65.25
**Our Method**	88.10	92.31	89.06	89.98	89.36	91.07
